# Paraspinal Extramedullary Hematopoiesis in a Transfusion-Dependent Beta-Thalassemia Patient: A Case Report

**DOI:** 10.7759/cureus.87271

**Published:** 2025-07-04

**Authors:** Zaina Rawashdeh, Ahmad Rawashdeh, Rama A Al-Rahamnah, Yara I Elghoul

**Affiliations:** 1 Faculty of Medicine, University of Jordan, Amman, JOR; 2 General Practice, Jordan University Hospital, Amman, JOR; 3 Hematology, Jordan University Hospital, Amman, JOR

**Keywords:** beta-thalassemia, extramedullary hematopoiesis, hydroxyurea, hypertransfusion, iron chelation, paraspinal mass, spinal cord compression

## Abstract

Extramedullary hematopoiesis (EMH) is a compensatory mechanism in chronic anemias, such as transfusion-dependent beta-thalassemia (TDT), most commonly affecting the liver and spleen. Paraspinal EMH is rare and may lead to spinal cord compression, resulting in neurological deficits. We present a 26-year-old male patient with longstanding TDT who developed progressive bilateral lower limb weakness, pelvic paresthesia, and acute urinary retention. MRI revealed a bilateral paraspinal mass compressing the dural sac and spinal cord, with concurrent severe myocardial and hepatic iron overload. Given the high operative risk and contraindications to surgery and radiotherapy, treatment included hypertransfusion, dual iron chelation therapy, and hydroxyurea. The patient developed hydroxyurea-induced bone marrow aplasia requiring discontinuation of treatment. Over six months, motor and sensory functions improved, bladder function partially recovered, iron markers decreased, and MRI showed regression of the mass. This case highlights the complexities in diagnosing and managing paraspinal EMH in high-risk TDT patients and supports the effectiveness of individualized conservative therapy.

## Introduction

Beta-thalassemia results from insufficient beta-globin production, leading to ineffective erythropoiesis and chronic anemia [1]. Transfusion dependency suppresses extramedullary hematopoiesis (EMH), the production of blood cells outside the bone marrow, which typically develops in the liver and spleen but may, on rare occasions, affect paraspinal regions, posing a risk of neurological compromise [2,3]. Paraspinal EMH occurs in less than 1% of transfusion-dependent beta-thalassemia (TDT) patients, compared to nearly 20% in non-transfusion-dependent thalassemia (NTDT) [4]. Magnetic resonance imaging (MRI) is the diagnostic method of choice for identifying paraspinal EMH [5]. Treatment options include transfusion, iron chelation, hydroxyurea, radiotherapy, or surgery, selected based on patient-specific risks and presentation [6]. In this report, we present a rare case of paraspinal EMH in a TDT patient, highlighting effective conservative management.

## Case presentation

**Figure 1:**
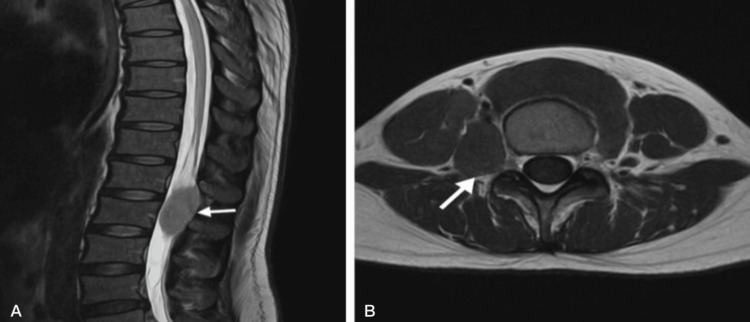
Sagittal and axial MRI of the lumbar spine showing a left paraspinal mass consistent with extramedullary hematopoiesis (A) Sagittal T2-weighted MRI of the lumbar spine shows a well-defined paraspinal soft tissue mass at the L4–L5 level (white arrow), causing mild compression of the dural sac and anterior displacement of the spinal cord. (B) Axial T2-weighted MRI confirms the presence of a left paraspinal mass that is isointense to mildly hyperintense relative to adjacent muscle (white arrow), without evidence of vertebral destruction or spinal canal invasion. These imaging features are consistent with extramedullary hematopoiesis in a patient with transfusion-dependent beta-thalassemia.

A 26-year-old male with transfusion-dependent beta-thalassemia (TDT) presented with a three-month history of progressive bilateral lower limb weakness, numbness, pelvic paresthesia, and acute urinary retention. He denied any additional symptoms or history of trauma and was not taking any regular medications. Neurologic examination revealed spastic paraparesis with motor strength graded 3/5, hyperreflexia, and diminished sensation below the T10 dermatome. Upon admission, the patient’s vital signs were stable, including a body temperature of 36.8 °C, blood pressure of 115/75 mm Hg, respiratory rate of 18 breaths/minute, heart rate of 85 bpm, and oxygen saturation of 97% on room air (fraction of inspired oxygen (FiO₂): 21%). Post-void residual volume was measured at 500 mL. Laboratory investigations revealed hemoglobin 7.5 g/dL, ferritin 4,000 ng/mL, reticulocyte count 2.1%, and white blood cell count 6.5 × 10³/μL. Cardiac and hepatic T2* MRI demonstrated severe iron overload, with cardiac T2* of 8 ms and liver T2* of 6 ms. Lumbar spine MRI demonstrated a bilateral paraspinal soft tissue mass extending from L4 to L5. The mass markedly compressed the dural sac and displaced the spinal cord anteriorly (Figure 1). Differential diagnoses included neurogenic tumors and epidural abscess, which were excluded based on imaging features and clinical context.

Differential diagnoses, including neurogenic tumors and epidural abscess, were ruled out based on both radiological characteristics and the patient’s clinical background. The lesion appeared bilateral, lobulated, and sharply defined, demonstrating intermediate signal intensity on T1-weighted MRI and low signal on T2-weighted images, findings that are typically associated with extramedullary hematopoiesis (EMH), especially in patients with transfusion-dependent hemoglobinopathies. Furthermore, the absence of features commonly seen in peripheral nerve sheath tumors, such as nerve root involvement, cystic changes, or significant contrast enhancement, further favored the diagnosis of EMH. Although peripheral nerve sheath tumors were a consideration, their imaging hallmarks, such as contrast enhancement or cystic change, were absent in this case. Given the patient’s long-standing history of TDT, severe systemic iron overload, hepatosplenomegaly, and the high-risk location of the mass, a biopsy was not pursued. Management was started empirically, drawing on published reports demonstrating successful reduction of EMH masses through hypertransfusion and iron chelation [7]. Subsequent clinical and radiologic improvement retrospectively confirmed the working diagnosis.

Due to the high surgical risk related to severe iron overload and contraindications to radiotherapy, the patient was managed conservatively. A hypertransfusion regimen was initiated, targeting a pre-transfusion hemoglobin level of >9.5 g/dL. Dual chelation therapy with oral deferasirox (30 mg/kg/day) and subcutaneous deferoxamine (40 mg/kg, five days per week) was administered to reduce the iron burden at moderate doses, to decrease the side effects, and to prevent the potential toxicity associated with combination therapy. Hydroxyurea (15 mg/kg/day) was added to suppress ineffective erythropoiesis.

**Figure 2:**
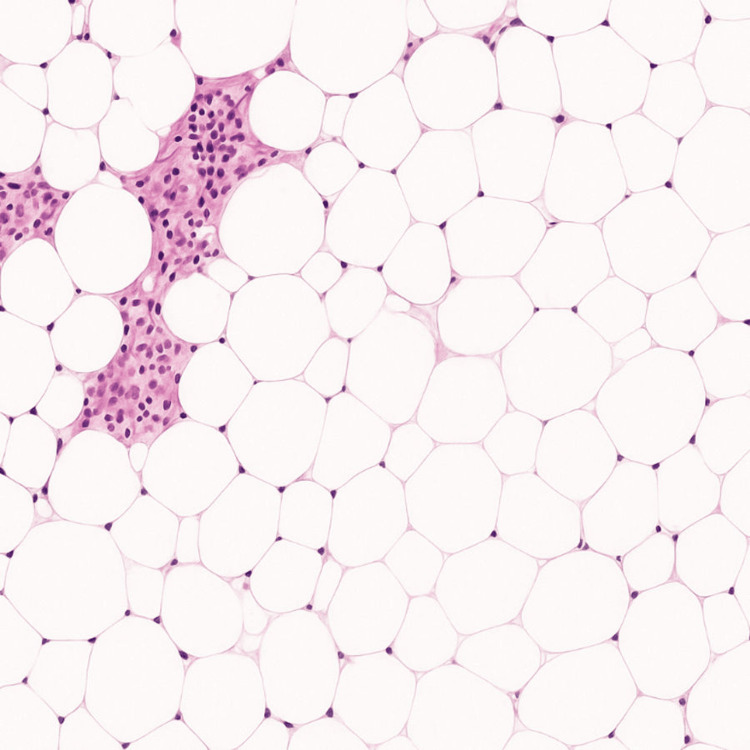
Bone marrow biopsy histopathology showing hypocellularity with predominant adipose tissue, suggestive of marrow aplasia likely secondary to chronic transfusion therapy in beta-thalassemia Histopathology of the bone marrow biopsy demonstrating marked hypocellularity with extensive adipocyte infiltration and reduced hematopoietic elements. These findings are consistent with drug-induced marrow aplasia, likely secondary to hydroxyurea therapy in transfusion-dependent beta-thalassemia patient.

After two months of therapy, the patient developed pancytopenia as a side effect of hydroxyurea rather than hypersplenism due to splenomegaly not being detected. Bone marrow biopsy confirmed drug-induced aplasia, prompting the discontinuation of hydroxyurea (Figure 2). A biopsy was deferred due to the lesion’s radiological consistency with EMH, the patient’s known thalassemia background, and the high-risk anatomical location. Over the following six months, the patient’s motor strength improved to 4/5, sensory deficits resolved, and bladder function partially recovered. Ferritin levels declined to 1,800 ng/mL, and cardiac T2* improved from 8 ms to 12 ms, reflecting partial reversal of iron overload. Follow-up spinal MRI demonstrated significant regression of the paraspinal mass and decompression of the spinal cord (Table 1). Regression of EMH masses with transfusional therapy has been demonstrated in prior reports, supporting its use in high-risk patients where biopsy and surgery are contraindicated [8].

**Table 1:** Patient’s laboratory parameters on admission, at two months (following hydroxyurea discontinuation), and at the six-month follow-up

Parameter	On Admission	2 Months (Hydroxyurea Discontinued)	6 Months Follow-up	Reference Range
Hemoglobin (g/dL)	7.5	>9.5	>9.5	13 – 17
Ferritin (ng/mL)	4000	N/A	1800	20 – 300
Reticulocyte count (%)	2.1	N/A	N/A	0.5 – 2.5%
WBC (×10³/μL)	6.5	↓ (pancytopenia)	Recovered	4.0 – 11.0 ×10³/μL

## Discussion

EMH typically arises as a compensatory response in TDT patients. In the absence of a definitive biopsy, the diagnosis was supported by the patient’s treatment response and imaging regression. Although EMH is more commonly reported in NTDT, it can also arise in TDT, particularly when transfusion and iron chelation are suboptimal [9]. Paraspinal EMH masses, particularly in the thoracic spine where the spinal canal is narrow, can lead to cord compression and neurological deficits [10]. MRI plays a pivotal role in differentiating EMH from other spinal masses such as tumors or infections [11]. Conservative treatments, such as blood transfusion alone, have demonstrated reversal of cord compression by reducing ineffective erythropoiesis and EMH mass [12]. Hydroxyurea promotes fetal hemoglobin production and suppresses ineffective erythropoiesis but carries a risk of marrow suppression, as seen in this case [13]. Radiotherapy is effective in rapidly reducing EMH mass and relieving symptoms, particularly when surgery is contraindicated; however, a relapse is possible, and long-term effects on spinal cord integrity remain a concern. Mass regression was anticipated based on literature showing that reducing ineffective erythropoiesis through hypertransfusion decreases EMH burden [14]. Surgical decompression is considered in severe or refractory cases but carries high risks in patients with significant iron overload and comorbidities [15]. A similar case reported spinal cord compression due to EMH successfully managed without surgery or radiotherapy, reinforcing the viability of conservative therapy in comparable clinical settings [16].

Given the diagnostic challenges and therapeutic risks, multiple case reports have demonstrated that paraspinal EMH can be reliably diagnosed based on imaging and clinical background alone in high-risk patients, particularly when biopsy poses a significant risk [17]. Our patient’s clinical improvement following hypertransfusion, aggressive iron chelation, and temporary hydroxyurea underscores the potential of individualized conservative therapy in high-risk patients. This approach avoids the complications associated with surgery and radiotherapy while achieving meaningful neurological recovery and radiological regression [18]. The absence of standardized guidelines, combined with a high operative risk and complications from medical therapy, poses a major challenge in managing paraspinal EMH in transfusion-dependent thalassemia.

## Conclusions

Paraspinal EMH is a rare but major complication in TDT that may lead to neurological impairments due to spinal cord compression, which necessitates early diagnosis and customized management. Timely MRI identification provides accurate diagnosis and unnecessary invasive treatment. Individualized conservative management, which may involve hypertransfusion, iron chelation, and hydroxyurea as indicated, can lead to significant clinical and radiological improvement, even in high-risk patients. In our case, histopathological evaluation prompted the discontinuation of hydroxyurea, highlighting the importance of regular monitoring of treatment-related toxicities, which remains essential for optimizing outcomes to ensure the safe implementation of conservative treatment.

## References

[1] Rund D, Rachmilewitz E (2005). β-thalassemia. N Engl J Med.

[2] Hassanzadeh M (2013). Images in clinical medicine. Extramedullary hematopoiesis in thalassemia. N Engl J Med.

[3] Haidar R, Mhaidli H, Taher AT (2010). Paraspinal extramedullary hematopoiesis in patients with thalassemia intermedia. Eur Spine J.

[4] Karimi M, Cohan N, De Sanctis V, Mallat NS, Taher A (2014). Guidelines for diagnosis and management of beta-thalassemia intermedia. Pediatr Hematol Oncol.

[5] Musallam KM, Taher AT, Rachmilewitz EA (2012). β-thalassemia intermedia: a clinical perspective. Cold Spring Harb Perspect Med.

[6] Algiraigri AH, Wright NA, Kassam A (2014). Hydroxyurea for β-thalassemia major: a meta-analysis. Blood.

[7] Emamhadi M, Alizadeh A (2012). Effect of hypertransfusion on extramedullary hematopoietic compression mass in thalassemia major: a case report. Iran J Radiol.

[8] Munn RK, Kramer CA, Arnold SM (1998). Spinal cord compression due to extramedullary hematopoiesis in beta-thalassemia intermedia. Int J Radiat Oncol Biol Phys.

[9] A Subahi E, Ata F, Choudry H (2022). Extramedullary haematopoiesis in patients with transfusion dependent β-thalassaemia (TDT): a systematic review. Ann Med.

[10] Olivieri NF, Brittenham GM (1997). Iron-chelating therapy and the treatment of thalassemia. Blood.

[11] Chaljub G, Guinto FC Jr, Crow WN, Kumar R (1991). MRI diagnosis of spinal cord compression in beta-thalassemia. Spine (Phila Pa 1976).

[12] Lee AC, Chiu W, Tai KS, Wong V, Peh WC, Lau YL (1996). Hypertransfusion for spinal cord compression secondary to extramedullary hematopoiesis. Pediatr Hematol Oncol.

[13] Cario H, Wegener M, Debatin KM, Kohne E (2002). Treatment with hydroxyurea in thalassemia intermedia with EMH masses. Ann Hematol.

[14] Malik M, Pillai LS, Gogia N, Puri T, Mahapatra M, Sharma DN, Kumar R (2007). Paraplegia due to extramedullary hematopoiesis in thalassemia treated successfully with radiation therapy. Haematologica.

[15] (2021). 2021 Guidelines for the Management of Transfusion‑Dependent β‑Thalassaemia (TDT). 4th Edn. Nicosia, Cyprus: Thalassaemia International Federation.

[16] Alorainy IA, Al‑Asmi AR, del Carpio R (2000). MRI features of epidural extramedullary hematopoiesis. Eur J Radiol.

[17] Tai SM, Chan JS, Ha SY, Young BW, Chan MS (2006). Successful treatment of spinal cord compression secondary to extramedullary hematopoietic mass by hypertransfusion in a patient with thalassemia major. Pediatr Hematol Oncol.

[18] La VT, Diatte M, Gaston J, Dick D, Sweiss R, Pakbaz Z (2018). Spinal cord compression due to extramedullary hematopoiesis in a patient with E-beta-thalassemia managed without radiation or surgery. J Community Hosp Intern Med Perspect.

